# First person – Annalisa Pinsino and Andi Alijagic

**DOI:** 10.1242/bio.042242

**Published:** 2019-03-15

## Abstract

First Person is a series of interviews with the first authors of a selection of papers published in Biology Open, helping early-career researchers promote themselves alongside their papers. Annalisa Pinsino and Andi Alijagic are co-first authors on ‘Sea urchin *Paracentrotus lividus* immune cells in culture: formulation of the appropriate harvesting and culture media and maintenance conditions’, published in BIO. Annalisa is a Third-class Researcher in her lab at Consiglio Nazionale delle Ricerche, Istituto di Biomedicina e Immunologia Molecolare ‘A. Monroy’, Palermo, Italy, investigating probing safety of nanoparticles by outlining sea urchin innate signalling pathways *in vitro*. Andi is a PhD Student in the lab of Dr Annalisa Pinsino.

**Annalisa and Andi at the 8th bi-annual ECOtoxicology meeting (BECOME 2018) in Livorno, Italy.**


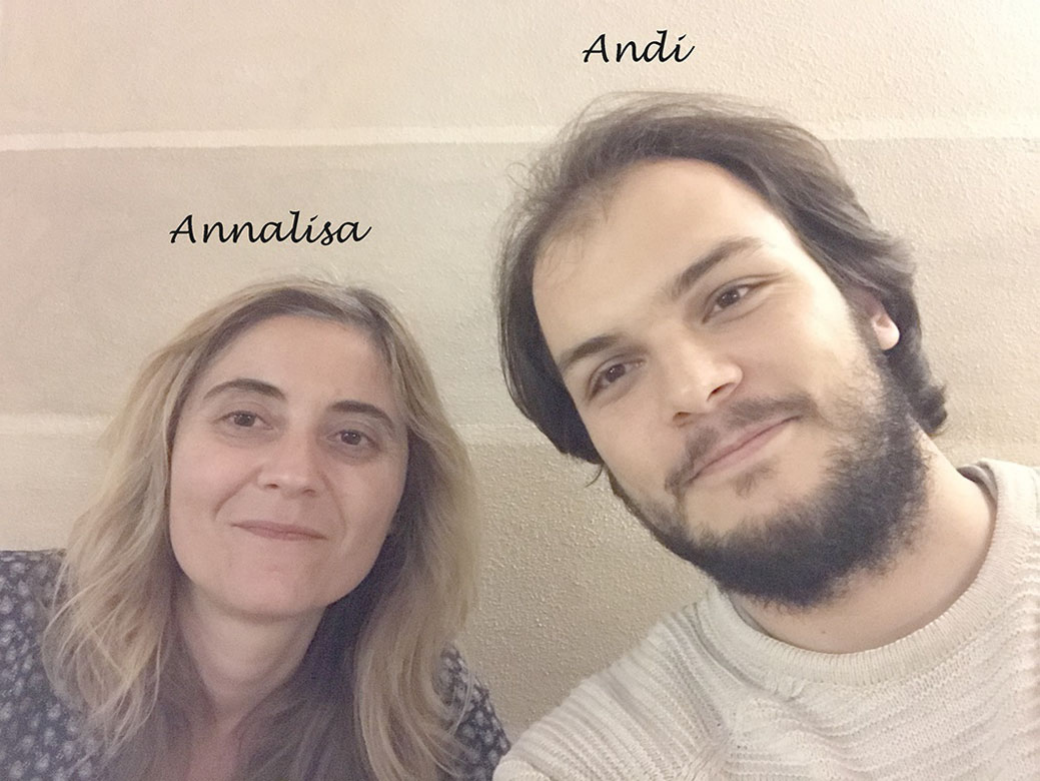


**What is your scientific background and the general focus of your lab?**

AP: I am a biologist, specialized in cell biology and development. My research is primarily dedicated to the molecular and cellular studies of cell regulation, signalling and differentiation mechanisms influencing development, life maintenance and immune response of a proxy to human adult/embryonic animal model (sea urchin). My current research focuses on understanding the potential immune signalling pathways involved in the persistent nanoparticles-immune cell interactions *in vitro*: from extracellular milieu to intracellular regulome. I am consolidating my independent programme in this field. Currently, I am a supervisory board member of the ETN PANDORA H2020 Marie Sklodowska-Curie ITN programme (GA 671881) and a scientific and training supervisory committee member of the DOC2AMU Nanoscreen H2020-MSCA-COFUND-2015 doctoral programme (GA 713750).

AA: I am a H2020 Marie Sklodowska-Curie PhD student (PANDORA programme) currently focused on in-depth interactions between sea urchin innate immunity and nanoparticles *in vitro*. As the recent research deciphering nano-bio interactions is tired of the predictive testing models, I am aiming my attention at the highly susceptible sea urchin's innate immune system in order to elucidate biological fate and impacts of nanoparticles on immune homeostasis.

**How would you explain the main findings of your paper to non-scientific family and friends?**

AP and AA: The sea urchin *Paracentrotus lividus* is a common echinoid living in the northeast Atlantic ocean and the Mediterranean sea, but its populations have recently collapsed and today it is an extremely rare species in some regions. Unfortunately, this species ─ which is an important food source for fishes and other animals, including humans ─ is facing increasing anthropogenic pressures in its coastal environments. Here we report a new method for the harvesting and maintenance of primary immune cells isolated from adult *P. lividus*. Cell culture is the process by which a scientist grows and maintains cells under carefully controlled conditions outside of a living animal. The goals of our studies on sea urchin immune cell culture were to reduce the number of sea urchins used for experimentation, to minimise the need for *in vivo* studies and to refine the use of this model organism as a proxy to humans.

**What are the potential implications of these results for your field of research?**

AP and AA: The sea urchin *P. lividus* has great potential as an experimental resource because many mechanisms of the immune response are conserved across many organisms, including humans (Alijagic and Pinsino, 2017). Therefore, appropriate culture methods for sea urchin immune cells provide an invaluable and amenable model for answering immunological questions while limiting the use of mammalian organisms.

“...appropriate culture methods for sea urchin immune cells provide an invaluable and amenable model for answering immunological questions while limiting the use of mammalian organisms.”

**What has surprised you the most while conducting your research?**

AP and AA: It was an encouraging surprise to see that the medium that has to be used to successfully culture *P. lividus* immune cells is not the basal nutrient medium used for a wide variety of mammalian cell lines and cell types. In the case of sea urchin *in vitro* culture it is not necessary to supply medium with all essential nutrients for the cells (e.g. vitamins, amino acids, lipids, nucleic acid precursors, carbohydrates, trace elements). On the contrary, we had to exploit the nature of the coelomic fluid, in which immune cells reside and move, rich of essential trophic and activating factors produced by immune cells.

**Figure:**
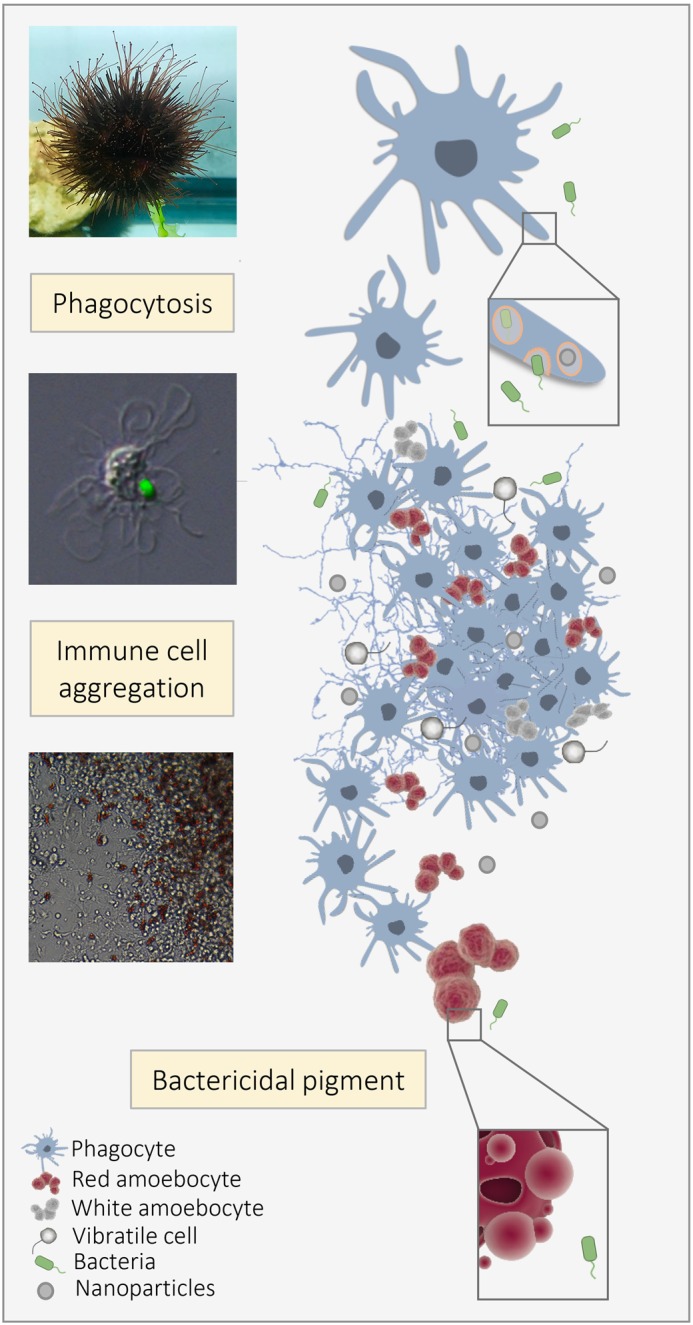
**Sea urchin model to study immunity for the safe use of nanomaterials.**

**What, in your opinion, are some of the greatest achievements in your field and how has this influenced your research?**

AP and AA: For over a century, the sea urchin has worked as a model organism in developmental biology research, contributing to understand the signalling that controls cell fitness during embryonic development and the organization and function of developmental Gene Regulatory Network. Analyses of the sea urchin genome revealed the close genetic relationship between sea urchin and humans, an extraordinary example of innate complexity and of non-self-recognition molecule diversity (Sea Urchin Genome Sequencing Consortium, 2006), thus further reinforcing the relevance of this model organism.

**What changes do you think could improve the professional lives of early-career scientists?**

AP: In my opinion, a serious reform of the current system of the financial support, permitting preferential treatment to budding researchers instead of to established researchers with the high track records, is required. Alternative funding models might genuinely improve the prospects of younger scientists, based upon the principal of ‘fund a project, not people's score, not people's age, not country's origin, not laboratory's fame’.

**What's next for you?**

AP: Studies on sea urchin resistance to immune and age-related diseases, cancer and tolerance to anthropogenic insults (e.g. nanomaterials) *in vitro*, may contribute to highlight the key protective molecules, which could be used in innovative applications at the cutting edge of biomedicine. In my opinion, an ambitious goal could be to reveal the functional identity of sea urchin immune cells and to provide intriguing suggestion concerning the possibility of the common origins and functions between sea urchin immune cells and a few ‘rare’ human immune cells recognized as an evolutionary bridge between innate and adaptive immunity.

## References

[BIO042242C1] Pinsino A. and Alijagic A. (2019). Sea urchin *Paracentrotus lividus* immune cells in culture: formulation of the appropriate harvesting and culture media and maintenance conditions. *Biology Open* 8, bio039289 10.1242/bio.039289 30718227 PMC6451355

